# *miR-9* upregulation leads to inhibition of erythropoiesis by repressing FoxO3

**DOI:** 10.1038/s41598-018-24628-0

**Published:** 2018-04-25

**Authors:** Yunyuan Zhang, Liping Li, Chunjie Yu, Vitalyi Senyuk, Fuxing Li, John G. Quigley, Tongyu Zhu, Zhijian Qian

**Affiliations:** 1 Department of Clinical laboratory, The Affiliated Hospital of Qingdao University Medical College, Qingdao, 266003 China; 2 Department of Medicine and Cancer Research Center, University of Illinois Hospital and Health Sciences System, Chicago, IL USA; 3 Department of Pediatrics, Tongji Hospital, Tongji University School of Medicine, Shanghai, China; 4 Fudan University ZhongShan Hospital, Shanghai, China

## Abstract

MicroRNAs (miRNAs) are emerging as critical regulators of normal and malignant hematopoiesis. In previous studies of acute myeloid leukemia miR-9 overexpression was commonly observed. Here, we show that ectopic expression of *miR-9 in vitro* and *in vivo* significantly blocks differentiation of erythroid progenitor cells with an increase in reactive oxygen species (ROS) production. Consistent with this observation, ROS scavenging enzymes, including superoxide dismutase (Sod2), Catalase (Cat), and glutathine peroxidase (Gpx1), are down-regulated by miR-9. In addition, *miR-9* suppresses expression of the erythroid transcriptional regulator FoxO3, and its down-stream targets Btg1 and Cited 2 in erythroid progenitor cells, while expression of a constitutively active form of FoxO3 (FoxO3-3A) reverses *miR-9*-induced suppression of erythroid differentiation, and inhibits *miR-9*-induced ROS production. Thus, our findings indicate that aberrant expression of *miR-9* blocks erythropoiesis by deregulating FoxO3-mediated pathways, which may contribute to the ineffective erythropoiesis observed in patients with hematological malignancies.

## Introduction

Over the last decade it has become apparent that non-coding RNAs function as regulators of crucial cellular processes and play a critical role in human diseases^1^. MicroRNAs are a class of evolutionally conserved, single-stranded, small (approximately 19−23 nucleotides) non-coding RNAs^2,3^. Mature miRNAs regulate gene expression by inducing targeted mRNA degradation, translational inhibition or through sequestration of targeted mRNA from the translational machinery^4^. Studies indicate that microRNAs are key regulators of normal hematopoiesis^5^ and deregulation of microRNAs has been implicated in the pathogenesis of hematological malignancies^6^. *MiR-9* regulates normal neurogenesis during brain development^7–9^. Notably, *miR-9* acts as a tumor suppressor and oncogene in a cell-context dependent manner^10^. The upregulation of miR9 expression has been noted in various hematological malignancies, including Hodgkin’s lymphoma^11^, AML^12–14^, ALL^15^ and MLL-AF9-induced leukemia^16^ while it also appears to be down-regulated in some cases of ALL^17^ and in pediatric AML with t(8;21)^18^. Anemia occurs frequently in patients with hematologic malignancies, such as acute leukemia and myelodysplastic syndromes, and is caused by a variety of mechanisms^19^. The role of miR-9 in erythropoiesis has not been reported previously.

In this study, we find that forced expression of *miR-9* inhibits erythroid progenitor differentiation both *in vitro* and *in vivo*, FoxO3 is a critical regulator of erythropoiesis and is required for regulation of oxidative stress in erythroid progenitor cells^20,21^. We observe that ectopic expression of *miR-9* induces reactive oxygen species (ROS) production in erythroid progenitor cells and inhibits their differentiation through regulation of FoxO3 expression. Our findings suggest that a low level of *miR-9* expression in erythroid progenitor cells is critical for maintenance of normal erythropoiesis.

## Results

### Forced expression of *miR-9* inhibits erythroid differentiation *in vitro*

**Figure 1:**
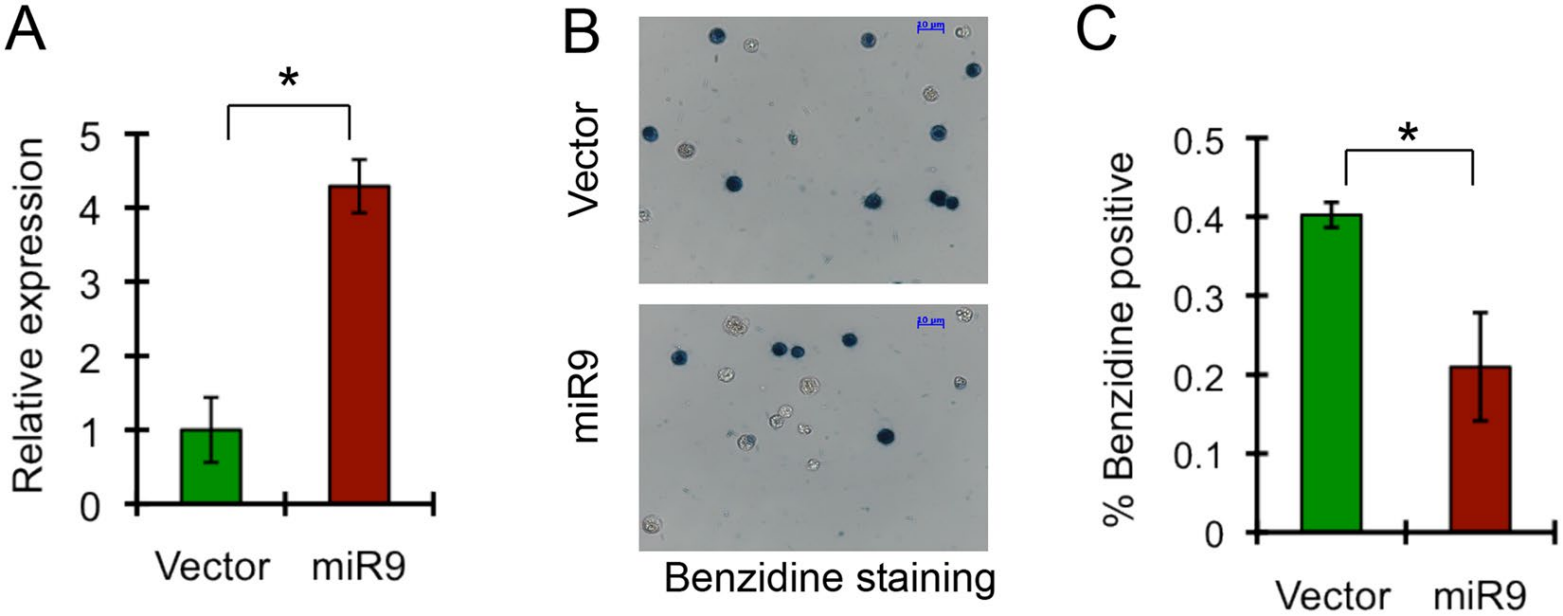
Forced expression of *miR-9* inhibits G1ER cell differentiation. (**A**) qRT-PCR analysis of *miR-9* precursor expression in Migr1 and Migr1-*MiR-9* transduced G1ER cells. Hprt gene was used for normalization. (**B**) Representative images of the benzidine staining of GFP^+^ G1ER cells expressing EGFP or *miR-9*/EGFP. Differentiation of the cells was induced with β-estrodial for two days. (**C**) The diagram shows the average percentage of Benzidine positive cells in GFP^+^ G1ER cells infected with control vector and *miR-9* in three independent experiments. *P < 0.05.

miR-9 overexpression has been noted in acute leukemia^12,15,16^. Previously, we showed that *miR-9* was highly expressed in mature myeloid cells but expressed at relatively low levels in erythroid cells^10^. We thus hypothesized that upregulation of *miR-9* may interfere with erythroid differentiation. We first assessed the consequences of *miR-9* overexpression on differentiation of G1ER cells. The G1ER cell line, derived from GATA-1 knockout murine embryonic stem cells, is widely used for analysis of erythroid differentiation *in vitro*. In this cell line, the coding region of GATA-1 is fused to the ligand-binding domain of the human estrogen receptor (ER). Exposure of G1ER cells to β-estradiol causes rapid activation of GATA-1 expression, inducing erythroid differentiation^22,23^. The *miR-9* precursor was subcloned into the retroviral vector Migr1, which expresses EGFP. Expression of *miR-9* in G1ER cells was confirmed by qRT-PCR (Fig. 1A). EGFP+ cells transduced by Migr1 or Migr1-*miR-9* were sorted, and then differentiation was induced by treatment with β-estradiol for 48 hours. Hemoglobin expression, determined by benzidine staining, is a hallmark of terminal erythroid differentiation. As shown in Fig. 1B,C, the percentage of benzidine positive cells was significantly lower in *miR-9*-transduced G1ER cells than in Migr1-transduced G1ER cells (20% in *miR-9*-transduced vs. 40% in Migr1-transduced), suggesting that ectopic expression of *miR-9* inhibits erythroid differentiation of G1ER cells.

**Figure 2:**
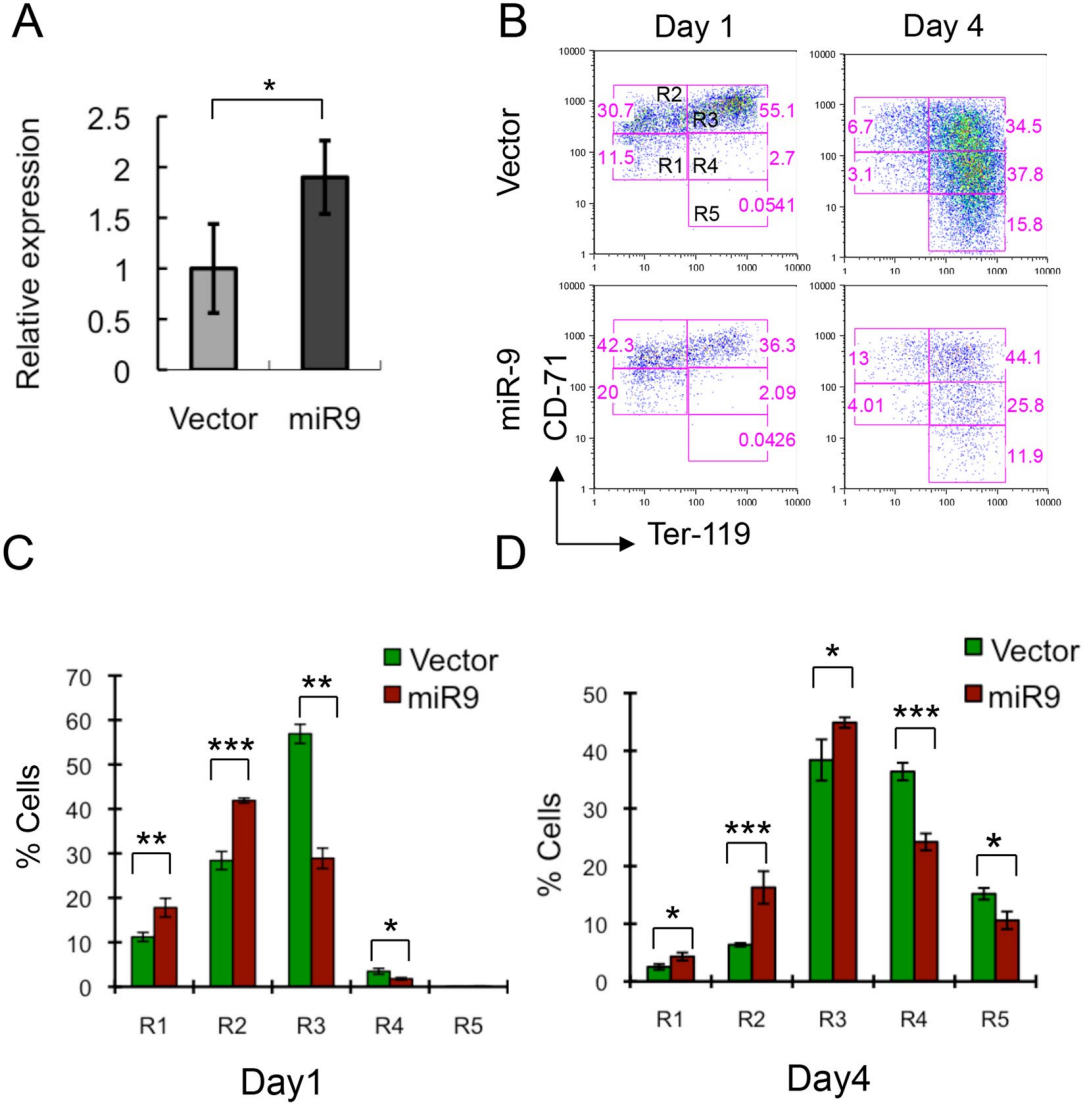
Ectopic expression of *miR-9* blocks differentiation of primary erythroblasts *in vitro*. (**A**) qRT-PCR analysis of *miR-9* expression in Migr1 and Migr1-*miR-9* transduced E14.5 fetal liver cells. (**B**) Representative histograms of flow cytometric analysis of erythroblasts at day 1 and day 4 after infection with vector and *miR-9* retrovirus. The switch from growth medium to differentiation medium at day 2 promoted erythroblasts differentiation. (**C**,**D**) Histograms of average ratio of R1, R2, R3, R4 and R5 in erythroblasts at day 1 and day 4. The experiments were performed in triplicate and repeated three times. *P < 0.05; **P < 0.01; ***P < 0.001.

As the terminal differentiation *in vitro* of erythroblasts derived from fetal liver closely mimics erythroid cell differentiation *in vivo*^24^, we next examined whether ectopic expression of *miR-9* affects the erythroid differentiation of fetal liver erythroblasts. In fetal liver cells, five distinct populations of cells, corresponding to erythroblasts at different developmental stages, R1 (CD71^med^Ter119^low^), R2 (CD71^high^Ter119^low^), R3 (CD71^high^Ter119^high^), R4 (CD71^med^Ter119^high^), and R5 (CD71^low^Ter119^high^), can be identified by their expression of characteristic cell surface markers. R1 cells are the least differentiated, while the R2-R5 populations are progressively more differentiated^24^. Ter119^**−**^ fetal liver cells were isolated from E14.5 embryos and infected with retrovirus expressing either Migr1 or Migr1-*MiR-9*. We observed that retroviral transduction of fetal liver cells with Migr1-*MiR-9* results in a 2-fold increase in *miR-9* expression (Fig. 2A). We then performed flow cytometric analysis on gated GFP-positive cells. One day after retrovirus infection, the majority of erythroblasts were in the early development stages R1, R2, and R3 (left panels, Fig. 2B,C). The proportion of R1 and R2 populations in the Migr1-*miR-9* transduced erythroblasts was significantly higher than that observed in the Migr1-transduced erythroblasts; whereas, the proportions of R3 and R4 populations were significantly reduced in the Migr1-*miR-9* transduced erythroblasts compared to Migr1-transduced erythroblasts (left panel, Fig. 2B,C). Erythroblasts were induced to differentiate at day 2 after infection. Three days after induction of differentiation (day 4), the majority of erythroblasts were differentiated (comprising R3, R4, and R5 populations). Consistently, the proportion of cells in the R1, R2, and R3 populations were higher in Migr1-*miR-9* transduced erythroblasts than was observed in the Migr1-transduced erythroblasts; whereas, the proportions of cells in the R4 and R5 populations were significantly lower in Migr1-*miR-9* transduced erythroblasts than in the Migr1-transduced erythroblasts (Right panel, Fig. 2B,D). In summary, these studies suggest that *miR-9* overexpression inhibits differentiation of fetal liver-derived erythroblasts *in vitro*.

**Figure 3:**
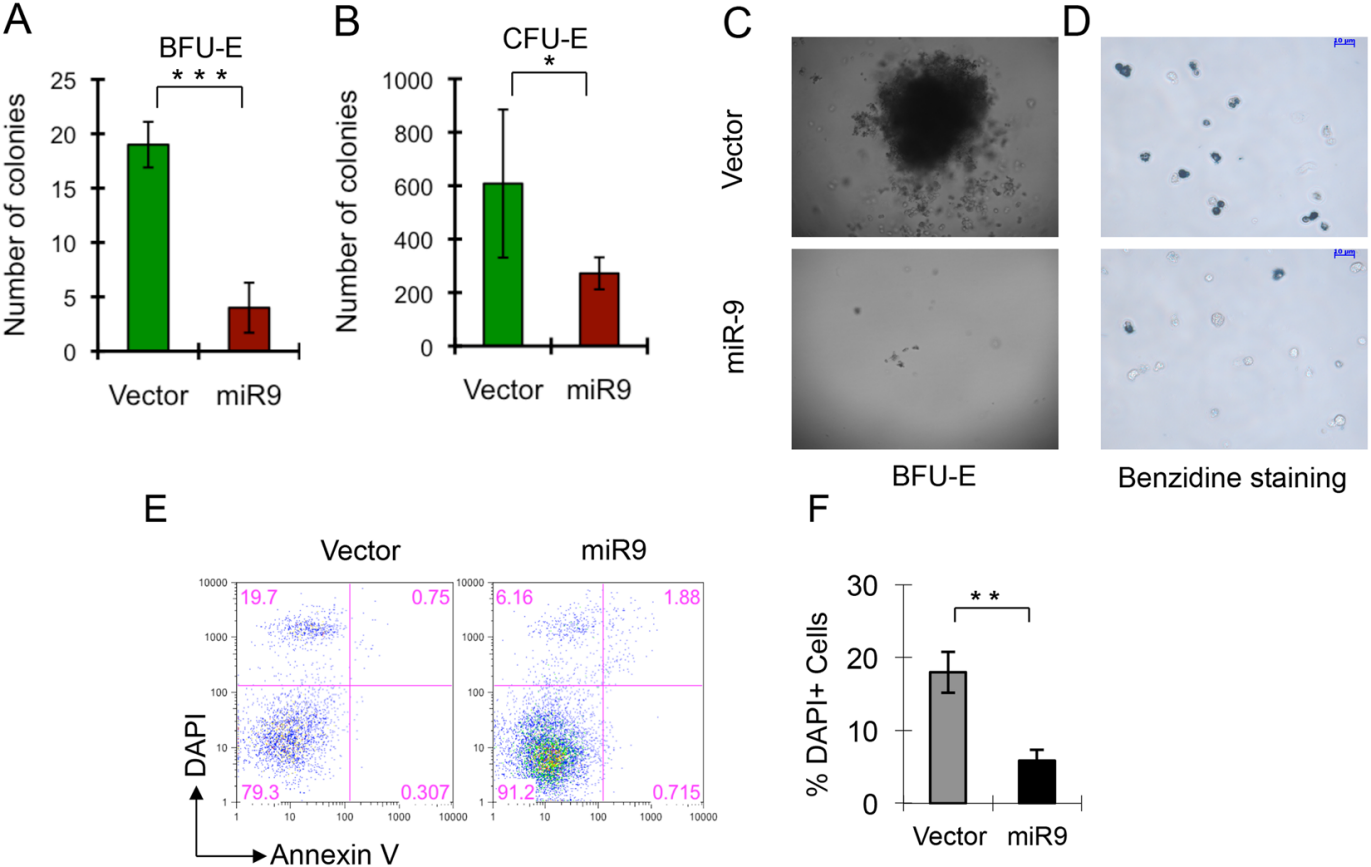
Forced expression of *miR-9* inhibits differentiation and reduces the frequency of cell death of early erythroid progenitor cells. Day 14.5 fetal liver cells were infected with Migr1 vector or Migr1-*miR-9*, and EGFP+ cells sorted and plated on methylcellulose medium. (**A**) Histograms showing the number of BFU-E colonies. (**B**) Histograms showing the number of CFU-E colonies. (**C**) The representative BFU-E colonies formed by Vector and *MiR-9* infected fetal liver cells. (**D**) Representative image of benzidine staining of cells from BFU-E colonies. *P < 0.05; ***P < 0.001. (**E**) Representative histograms of flow cytometric analysis of apoptotic and dead cells in vector or miR-9 infected(GFP+) fetal liver cells two days after induction of differentiation *in vitro*. (**F**) The percentage of DAPI+ cells in vector or miR-9 infected fetal liver cells. Experiments were repeated three times. **P < 0.01.

In the erythroid lineage, burst-forming unit-erythroid (BFU-E) is recognized as the earliest committed progenitor, which further differentiates into colony-forming unit-erythroid (CFU-E). Upon stimulation by erythropoietin (Epo), CFU-E progenitor cells develop into erythoblasts^25,26^. To further evaluate the effects of *miR-9* overexpression on the erythroid differentiation potential of fetal liver cells, we performed BFU-E and CFU-E assays using EGFP^**+**^ sorted fetal liver cells that had been transduced with either Migr1 or the Migr1-*miR-9* vector. Notably, *miR-9*-transduced fetal liver cells gave rise to 2-3-fold less BFU-E and CFU-E colonies as compared to Migr1-transduced fetal liver cells (Fig. 3A,B). The *miR-9*-transduced BFU colonies also appeared much smaller than Migr1-transduced BFU colonies (Fig. 3C) and there was a significant reduction in the number of mature erythroid cells, as determined by benzidine staining (Fig. 3D). To determine whether the reduced BFU and CFU colony-forming ability of *miR-9*-transduced fetal liver cells is a consequence of increased cell death or apoptosis of these cells, we analyzed the status of cell death and apoptosis in both vector or *miR-9*-transduced fetal liver cells. Surprisingly, forced expression of miR-9 reduced the frequency of cell death (DAPI^+^) of fetal liver cells, but had a marginal effect on early apoptosis (Annexin V^+^, DAPI^−^) (Fig. 3E–F). Together, these results suggest that *miR-9* overexpression inhibits differentiation of erythroid progenitors.

### miR-9 blocks erythroid differentiation *in vivo*

**Figure 4:**
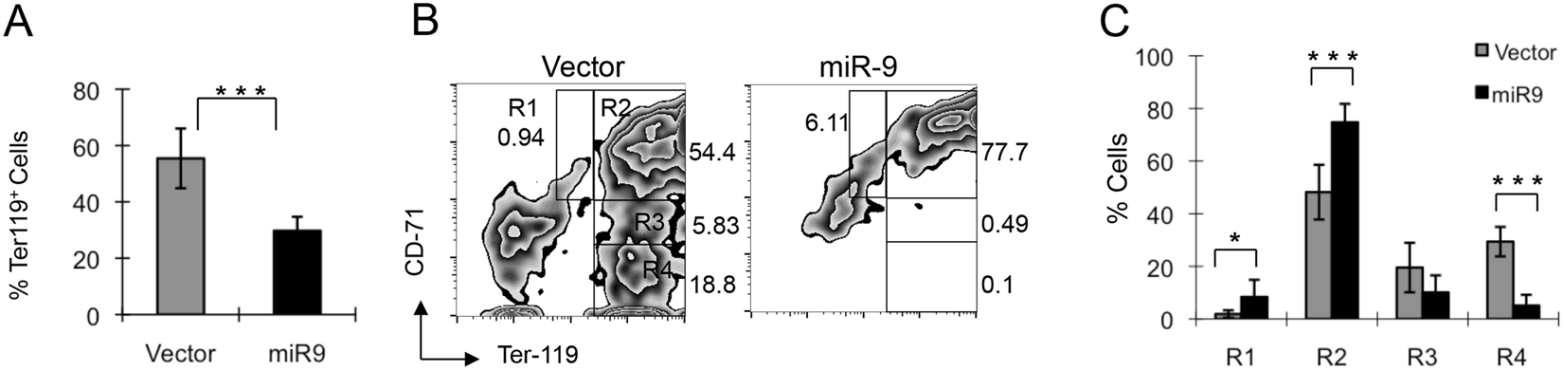
miR*-9* inhibits erythroblast cell differentiation *in vivo*. (**A**) The histogram shows the percentage of Ter119+ cells in gated EGFP+ cells in vector and *miR-9* chimeric mice. To generate the chimeric mice, the Lin^-^ primary bone marrow cells were infected with retrovirus expressing miR-9 or control vector, followed by transplanted into lethally-irradiated wildtype recipient mice. (**B**) Flow cytometric analysis of the frequencies of erythroblasts in bone marrow cells from the representative vector and chimeric mice. (**C**) Histogram shows the average frequencies of erythroblasts in vector and *miR-9* chimeric mice (means ± SD, n = 5–7). *P < 0.05; ***P < 0.001.

To evaluate whether ectopic expression of *miR-9* also affects erythroid differentiation *in vivo*, we generated chimeric mice reconstituted with BM cells expressing *miR-9*/EGFP or EGFP. Lin^**-**^ BM cells isolated from C57B/L6 mice were infected with Migr1 or Migr1-*miR-9* retrovirus, followed by transplantation into lethally-irradiated wildtype recipient mice. At 7 weeks post-transplantation, erythroid differentiation was examined using flow cytometric analysis of EGFP^+^ BM cells for CD71 and Ter119 cell-surface expression. In BM cells, erythroblasts can be separated into R1 (Ter119^−^CD71^+^), R2 (Ter119^+^CD71^+^), R3 (Ter119^+^CD71^med^), and R4 (Ter119^+^CD71^−^) populations, corresponding to proerythroblasts, basophilic erythroblasts, late basophilic and polychromatophilic erythroblasts, and orthochromatophilic erythroblasts, respectively. As shown in Fig. 4, the frequency of R1 and R2 erythroblasts was significantly higher in *miR-9*-EGFP^+^ transduced BM cells than it was in EGFP^+^ control transduced BM cells. In contrast, when compared to mice transplanted with control Migr1 cells, mice transplanted with *miR-9*-EGFP+ cells had a significantly lower frequency of late stage erythroblasts (R4 population, Ter119^+^, CD71^+^). As observed *in vitro*, these data indicate that miR9 overexpression blocks erythroid differentiation *in vivo*.

### MiR-9 regulates ROS production in erythroid progenitors

**Figure 5:**
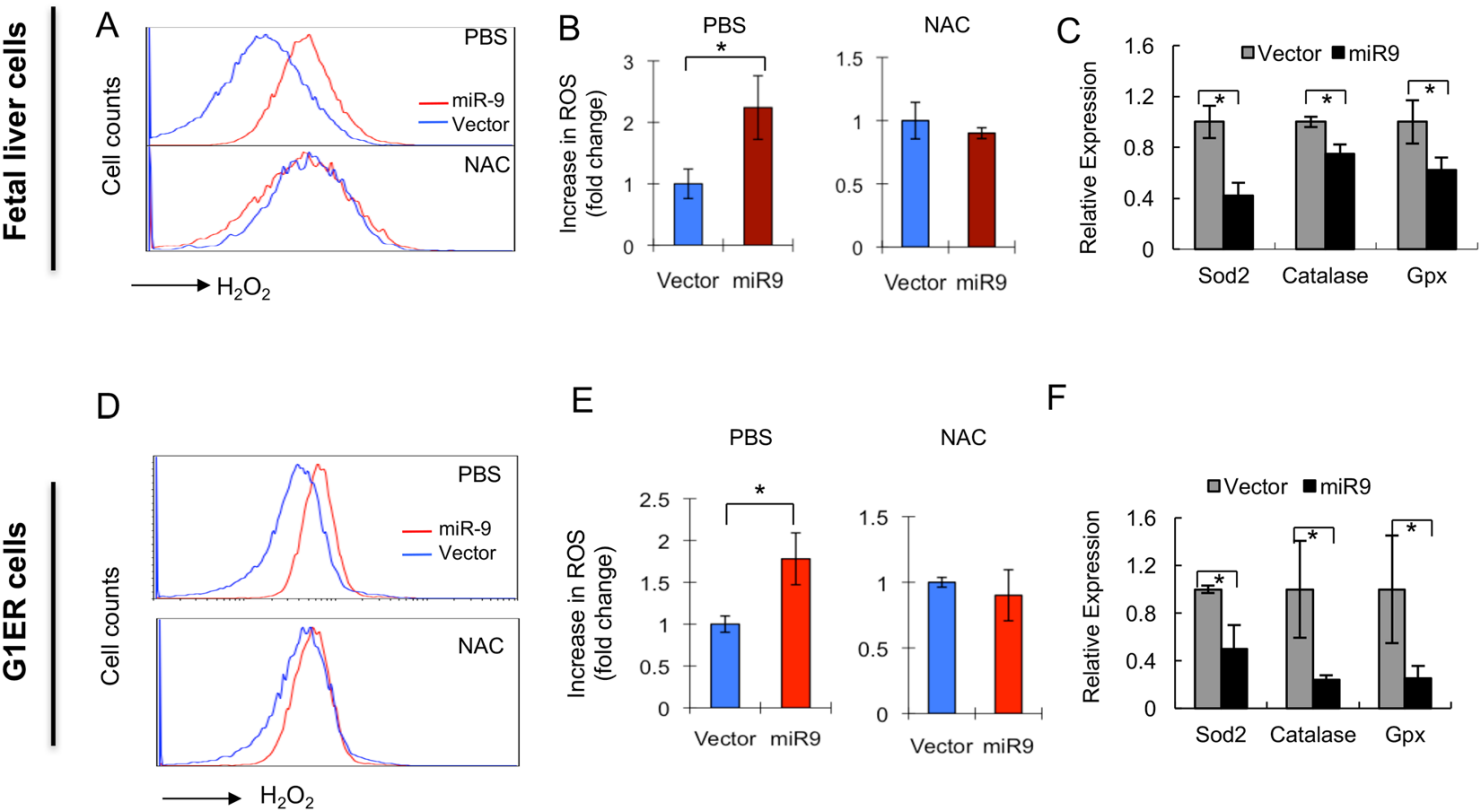
Ectopic expression of *miR-9* increases production of ROS in fetal liver cells and G1ER cells. (**A**) Representative histograms show ROS levels in GFP + fetal cells infected with Migr1 or Migr1-*miR-9*. Top panel, untreated cells; bottom panel, cells treated with NAC. (**B**) Summary of ROS levels in Migr1 and Migr1-*miR-9* transduced fetal liver cells without treatment (left panel) or with NAC treatment (right panel). (**C**) qRT-PCR analysis of the expression of ROS scavenging enzymes in vector or *miR-9* infected fetal liver cells. Experiments were repeated three times. (**D**) Representative histograms show ROS levels in GFP+G1ER cells infected with Migr1 or Migr1-miR-9. Top panel, untreated cells; bottom panel, cells treated with NAC. (**E**) Summary of ROS levels in Migr1 and Migr1-miR-9 transduced G1-ER cells without treatment (left panel) or with NAC treatment (right panel). (**F**) qRT-PCR analysis of the expression of ROS scavenging enzymes in vector or miR-9 infected G1ER cells. *P < 0.05.

Oxidative stress plays a critical role in the maturation of erythroid progenitors^27^. We next examined ROS production in fetal liver erythroid progenitors and G1ER cells expressing either control vector or *miR-9*. In fetal liver cells, *miR-9*-transduced erythroid progenitors had a two-fold increase in ROS levels compared with control cells. However, incubation with the ROS scavenger N-acetyl-L-cysteine (NAC) inhibited *miR-9*-induced ROS production (Fig. 5A,B). Similarly, in G1ER cells, forced expression of *miR-9* also significantly increases ROS production, which is inhibited by NAC (Fig. 5D,E). Scavenging enzymes have an important role in controlling cellular ROS levels. To determine whether ROS scavenging enzymes are reduced in *miR-9*-transduced erythroid progenitors, we performed qRT-PCR analysis of these genes in both vector and *miR-9*-transduced erythroid progenitors and G1ER cells. As shown in Fig. 5C,F, expression of superoxide dismutase (Sod2), catalase (Cat) and glutathine peroxidase (Gpx1) enzymes were all significantly down-regulated in *miR-9* transduced cells. Thus, the increased ROS levels observed in *miR-9*-transduced cells may result from decreased expression of ROS scavenging enzymes.

### miR-9 down-regulates FoxO3 in erythroid progenitors and co-expression of miR-9 and FoxO3 reverses miR-9 induced inhibition of erythroid differentiation

**Figure 6:**
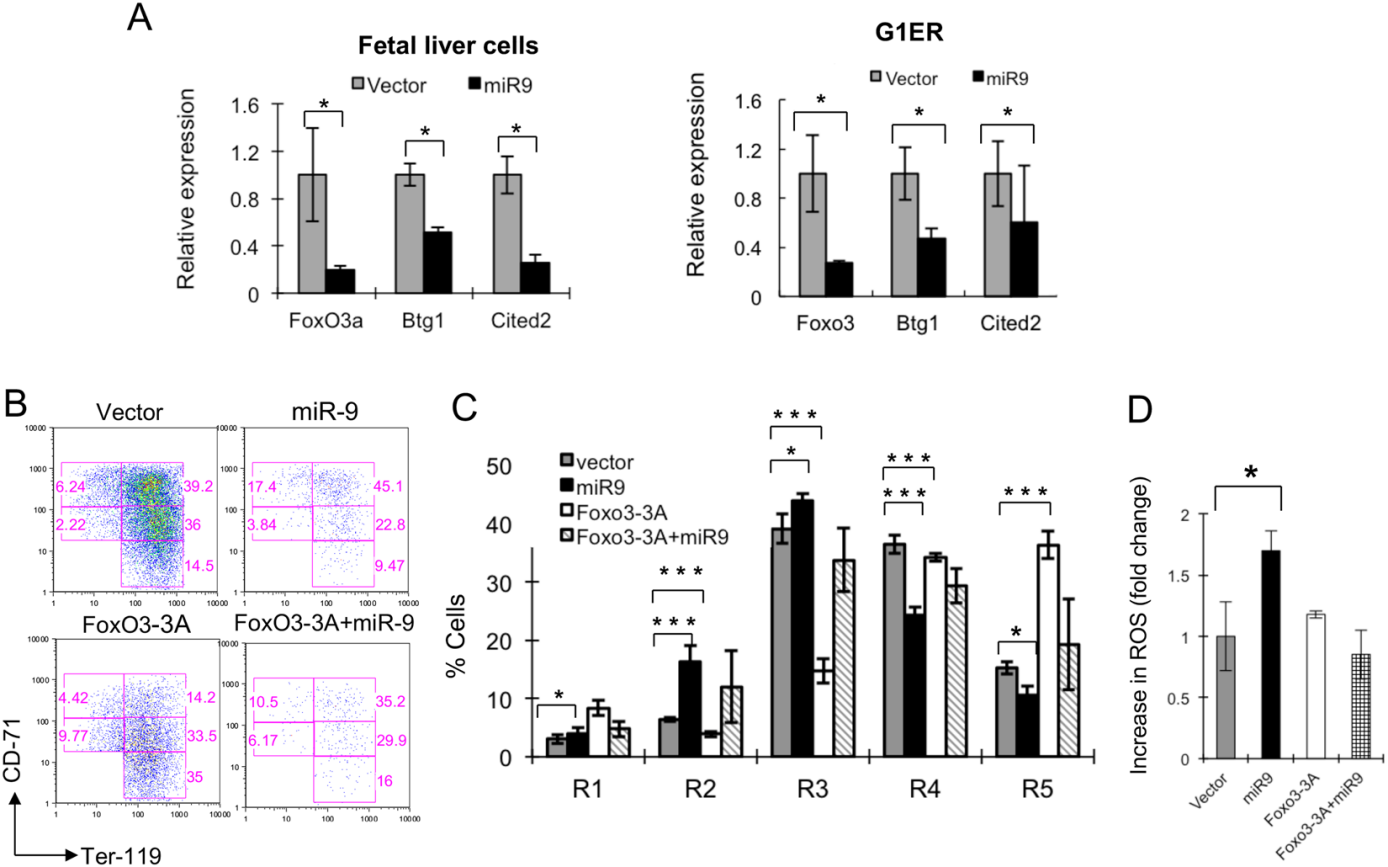
Forced expression of *FoxO3* reverses *miR-9*-mediated inhibition of erythroid progenitor cell differentiation and induction of ROS production. (**A**) qRT-PCR analysis of the expression of FoxO3, and its known down-stream targets in GFP+ fetal liver cells (left panel) and G1ER cells (right panel) transduced with vector or *miR-9*. (**B**) Representative histograms of flow cytometric analysis of GFP+ fetal liver cells transduced with Vector, *miR-9*, FoxO3-3A and FoxO3A/*miR-9*. (**C**) Histograms show the average percentage of cells in different stages of erythroid blast differentiation from these transduced fetal liver cells. (**D**) ROS levels in fetal liver cells transduced with vector, *miR-9*, FoxO3a and FoxO3a/*miR-9*. The experiment was performed in triplicate and repeated 2–3 times. *P < 0.05; ***P < 0.005.

Previously we demonstrated that *miR-9* down-regulates FoxO3 expression by directly binding the 3′ UTR region of FoxO3 in BM cells^10^. To determine whether *miR-9* inhibits FoxO3 expression in erythroid progenitors, we examined FoxO3 expression in *miR-9*- and vector-transduced fetal liver erythroid progenitors and G1ER cells. As determined by both Western Blot and qRT-PCR analysis, forced expression of *miR-9* significantly inhibits FoxO3 expression in both cell types (Fig. 6A, Supplementary Fig. 1). Concomitantly, Btg1 and Cited 2, known down-stream targets of FoxO3, were also markedly down-regulated in *miR-9*-transduced erythroid progenitors and G1ER cells as compared to vector-transduced cells (Fig. 6A). As FoxO3 is a known regulator of erythroid differentiation^21,28^, we hypothesized that the effects of miR9 on erythroid differentiation may be at least partially mediated by FoxO3. We therefore examined the induction of erythroid differentiation in fetal liver cells expressing either control vector, *miR-9*, a constitutively active form of FoxO3 (FoxO3-3A), or miR9 and FoxO3-3A. In line with a previous report^28^, forced expression of FoxO3-3A reduced the frequency of R2 and R3 erythroid blasts and increased the frequency of R5 (late) erythroid blasts. Notably, erythroid progenitors co-expressing *miR-9* and FoxO3-3A and control vector-transduced erythroid progenitors had comparable frequencies of erythroid blasts at both early and late stages (Fig. 6B,C), suggesting that constitutive expression of FoxO3 reverses the inhibitory effects of *miR-9* overexpression on erythroid progenitor differentiation.

### FoxO3-3A inhibits *miR-9*-induced ROS production

As FoxO3 regulates oxidative stress in erythropoiesis^28^, we determined whether FoxO3-3A suppressed the increase in ROS production induced by *miR-9* overexpression. Of note, ROS levels were comparable in erythroid progenitors expressing either control vector or both *miR-9* and FoxO3-3A vectors (Fig. 6D). Thus, it appears that the *miR-9*-induced increase in ROS levels is mediated by FoxO3 suppression.

## Discussion

Here, we find that ectopic expression of *miR-9* inhibits erythroid differentiation of G1ER cells and erythroid progenitor cells derived from murine fetal liver. Consistent with these results, our studies show that forced expression of *miR-9* through retroviral transduction of murine BM cells also significantly inhibits erythroid progenitor differentiation *in vivo*. In a previous study it was shown that *miR-9* directly binds the 3′ UTR region of the FoxO3 gene, suppressing its expression in myeloid progenitor cells^10^. Here we demonstrate that *miR-9* also downregulates FoxO3 mRNA and protein levels in erythroid progenitor cells, and that expression of FoxO3-3A, a constitutively active form of FoxO3, reverses *miR-9*-induced inhibition of erythroid differentiation. Thus, we provide the first evidence that *miR-9* regulates erythropoiesis via modulation of the FoxO3 pathway.

Importantly, *miR-9* has a previously unrecognized role in the regulation of ROS production during erythropoiesis. While ROS are normal byproducts of cell metabolism, excessive ROS production leads to oxidative stress that can damage biomolecules, and it is associated with hematopoietic malignancies^29^. Tight regulation of oxidative stress is critical for erythropoiesis and especially during maturation of erythrocytes as they accumulate hemoglobin^27,30^. Increased *miR-9* expression upregulates ROS production in erythroid progenitor cells, likely through downregulation of ROS scavenging enzymes such as Sod2, Cat and Gpx1. FoxO3 is an important regulator of oxidative stress during erythropoiesis, and loss of Foxo3 inhibits maturation of erythroid progenitors exposed to excessive oxidative stress^20,21^. Notably, we found that the constitutive mutant of FoxO3 (FoxO3-3A) reverses *miR-9*-induced ROS production. Thus, it appears that *miR-9* induces ROS production by dysregulating FoxO3-mediated mitigation of ROS stress.

miRNAs are known to have multiple downstream targets^31^. The function of *miR-9* is cell-context dependent, and likely mediated by a number of downstream pathways. For instance, *miR-9* regulates the development and function of neural progenitors by targeting genes including TLX^32^, FoxG1^33^ and Hes1^8,34^ while it induces metastasis of breast cancer cells by targeting LIFR and E-cadherin^35,36^. Although we demonstrate that FoxO3 mediates *miR-9* function in erythroid progenitor cells, our studies do not rule out the possibility that alternate *miR-9* downstream targets mediate other functions for *miR-9* during erythropoiesis.

## Methods

### Cell culture

Adherent 293 T cells were maintained in Dulbecco’s modified minimum essential medium (DMEM) supplemented with 10% fetal bovine serum (FBS). G1ER cells were maintained in Iscove’s modified Dulbecco’s medium containing 2% penicillin/streptomycin (Invitrogen), 2 units/ml erythropoietin, 120 nM monothioglycerol (Sigma), 0.6% conditioned medium from a Kit ligand-producing Chinese hamster ovary cell line and 15% fetal bovine serum (FBS). To induce differentiation, 100 nM beta-estradiol was added.

Fetal liver cells were mechanically isolated from E14.5-E15.5 C57B/L6 and cultured in erythroid-culture medium (StemPro-34, Invitrogen) containing 2 mM L-glutamine, 2% penicillin/streptomycin, 0.5 U/ml Epo, 1 uM dexamethasone and 100 ng/ml SCF.

### Retroviral constructs, Retroviral production and infection. Plasmids

The plasmids Migr1-*miR-9*, MSCV-puro-*miR-9*, Migr1-FoxO3-3A and Migr1-FoxO3-3a/*miR-9* were described in our previous study^10^. To generate infectious retrovirus particles, we transfected 293 T cells with retroviral vectors and pEco (ecotropic packaging plasmid) using MegaTrans 1.0 reagent (OriGene). After 24 h of cultivation, the fresh medium was replaced and virus were harvested every 12 h and used for subsequent experiments. G1ER cells, fetal liver cells or primary bone marrow cells were infected with retrovirus by spinoculation as described previously^37^.

### Erythroid differentiation *in vitro*

Total fetal cells were labeled with biotin-conjugated anti-Ter119 antibody, and the Ter119- cells were isolated by Dynabeads as per the manufacturer’s instructions (Dynabeads Biotin Binder, Invitrogen), followed by retroviral infection. Two days after infection, the cells were cultured in erythroid-culture differentiation medium (StemPro-34, Invitrogen) containing 2 mM L-glutamine, 2% penicillin/streptomycin, 10^−4▯^ M ▯β-mercaptoethanol, 0.5 mg/ml transferrin, 40 ng/ml IGF and 10 U/ml Epo.

### BM Transplantation

The donor BM cells were collected from C57BL/6 mice injected with 5-Fluorouracil (5-FU) intraperitoneally 5 days before collection. The recipient mice received a lethal dose of irradiation (10 Gy). After 12 hours, the mice were then administered 0.5–1.0 × 10^6^ retrovirally-infected BM cells by retroorbital injection. All of the animal studies were performed in accordance with the guidelines of the Animal Care Committee of the University of Illinois at Chicago and all procedures were approved by the Animal Care Committee of the University of Illinois at Chicago.

### Flow Cytometry Assays

Single-cell suspensions from BM were stained with the indicated fluorochrome-conjugated antibodies (eBioscience, USA). For the detection of apoptosis, fetal liver cells were stained with Anti-Annexin V antibody (eBioscience, USA) and DAPI. For the detection of cell cycle, fetal liver or G1ER cells were labeled with BrdU overnight, and stained with anti-BrdU-APC anibody (eBioscience, USA) and DAPI. For the ROS detection, fetal liver or G1ER cells were stained with hydroethidine (10 mM)(Molecular probe) following the manufacturer’s instructions. Flow cytometry was performed at the University of Illinois at Chicago core facility using CyAn flow cytometer. All data were analyzed by FlowJo software (TreeStar).

### Colony-Forming Assays

E14.5 D fetal liver cells were spinfected with the MSCV-puromycin retrovirus and the cells were selected by 1 μg/ml puromycin for 3 days. To detect CFU-E colonies, 1 × 10^4^ selected E14.5 fetal liver cells were plated in duplicate in semisolid medium (The Mouse Methylcellulose Complete Media; R&D systems) containing 4 U/ml erythropoietin according to the manufacturer’s protocol. To detect BFU-E colonies, 0.1 × 10^4^ selected E14.5 fetal liver cells were plated in duplicate in methylcellulose medium containing 4 U/ml erythropoietin and 100 ng/ml SCF. The CFU-E and BFU-E colonies were counted at 3 days or 14 days after plating respectively.

### qRT-PCR

Total RNAs isolated from fetal liver or G1ER cells were transcribed into cDNA using SuperScript III reverse transcriptase (Invitrogen), and subjected to qRT-PCR. The primers used were: Hprt, forward: 5′-gtaatgatcagtcaacgg-3′, reverse: 5′-ccagcaagcttgcaacct-3′; FoxO3, forward: 5′-acttcaaggataagggcgacagca-3′, reverse: 5′-cttcattctgaacgcgcatgaagc-3′, BTG1, forward: 5′-tggattacccaagtctgcaa-3′, reverse: 5′-caggaccgtagctgagtcaa-3′, Cited2, forward: 5′-cgcatcatcaccagcagcag-3′, reverse: 5′-cgctcgtggcattcatgttg-3′; SOD2, forward: 5′-gcctcccagacctgccttacga-3′, reverse: 5′-ggctgaagagcgacctgagttgta-3′, Catalyse, forward: 5′-gcacatgaatggctatggatca-3′, reverse: 5′-ttggcgatggcattgaaaagat-3′, Gpx, forward: 5′-tcgaacctgacatagaaaccct-3′, reverse: 5′-caccatcatggaagaaccg-3′; pre-*miR-9*–3, forward: 5′-ggaggcccgtttctctctttg-3′, reverse: 5′-gctttatgacggctctgtggc-3′.

### Western Blotting

The Western Blot was performed as described previously^38^. Rabbit FoxO3 antibody and Tubulin antibodies were obtained from Cell signaling technology and Millipore corporation respectively.

### Benzidine staining

Erythroid differentiation of G1ER and fetal liver cells was also evaluated by hemoglobin content. The concentration of hemoglobin was estimated by the standard methodology using benzidine staining. Staining solution was freshly prepared by adding 20 μl of 33% hydrogen peroxide to 1 ml of freshly prepared solution of 2% benzidine in 3% acetic acid (w/v). The solution was diluted 1:1 with the cell suspension. After 3 mins, the percentage of benzidine-positive cells (blue in color) was assessed under a light microscope. The percentage of positive cells was counted from a total of 200 cells. Each experiment was performed in triplicate.

### Statistical analysis

Statistical significance was calculated using the two tailed Student’s *t* test.

## Electronic supplementary material

supplementary dataset 1
