# Supplementary material for: miR-9 upregulation leads to inhibition of erythropoiesis by repressing FoxO3
Source: Sci Rep. 2018 Apr 25;8:6519. doi: 10.1038/s41598-018-24628-0 (PMC5916915; doi:10.1038/s41598-018-24628-0)
Supplement: Supplementary file 1 — supplementary dataset 1 [file 41598_2018_24628_MOESM1_ESM.docx]

***miR-9* upregulation leads to inhibition of erythropoiesis by repressing FoxO3**

*Yunyuan Zhang^1,2^, Liping Li^2,4^, Chunjie Yu^2^, Vitalyi Senyuk^2^, Fuxing Li ^2,3^, John G. Quigley^2^, Tongyu Zhu^4^, Zhijian Qian^2^**

*^1^ Department of Clinical laboratory, The Affiliated Hospital of Qingdao University Medical College, Qingdao 266003, China*

*^2^Department of Medicine and Cancer Research Center, University of Illinois Hospital and Health Sciences System, Chicago, IL;*

*^3^ Department of Pediatrics, Tongji Hospital, Tongji University School of Medicine, Shanghai, China*

*^4^Fudan University ZhongShan Hospital, Shanghai, China.*

**Supplementary Figure 1.  Forced expression of miR-9 inhibits Foxo3a expression in fetal liver cells and G1ER cells.** Western blot analysis of Foxo3 expression in GFP+ fetal liver cells (left panel) and G1ER cells (right panel) transduced with vector or miR-9
